# Serotonin Is a Key Factor for Mouse Red Blood Cell Survival

**DOI:** 10.1371/journal.pone.0083010

**Published:** 2013-12-17

**Authors:** Pascal Amireault, Elisa Bayard, Jean-Marie Launay, David Sibon, Caroline Le Van Kim, Yves Colin, Michel Dy, Olivier Hermine, Francine Côté

**Affiliations:** 1 Faculté de Médecine, Université Paris Descartes-Sorbonne Paris Cité, Institut Imagine, CNRS UMR 8147, Hôpital Universitaire Necker Enfants Malades, Paris, France; 2 Laboratoire d’excellence GR-Ex, Paris, France; 3 Inserm UMR_S665, Institut National de la Transfusion Sanguine, Paris, France; 4 Service de Biochimie, Assistance Publique-Hôpitaux de Paris, Hôpital Lariboisière, Paris, France; Universidade de Sao Paulo, Brazil

## Abstract

Serotonin (5-HT) is a monoamine originally purified from blood as a vasoactive agent. In nonneuronal tissues, its presence is linked with the expression of tryptophan hydroxylase 1 (TPH1) that catalyzes the rate-limiting step of its synthesis. Targeted disruption in mice of the *TPH1* gene results in very low levels of circulating 5-HT. Previous analysis of the TPH1 knockout (TPH1^−/−^) mouse revealed that they develop a phenotype of macrocytic anemia with a reduced half-life of their circulating red blood cells (RBC). In this study, to establish whether the observed reduced half-life of TPH1^−/−^ RBC is an intrinsic or an extrinsic characteristic, we compared their survival to RBC isolated from wild-type mice. Both *in vivo* and *in vitro* data converge to demonstrate an extrinsic protective effect of 5-HT since presence of 5-HT in the RBC environment protects RBC from senescence. The protective effect played by 5-HT is not mediated through activation of a classical pharmacological pathway as no 5-HT receptors were detected on isolated RBC. Rather, 5-HT acts as an effective antioxidant since reduction of 5-HT circulating levels are associated with a decrease in the plasma antioxidant capacity. We further demonstrate a link between oxidation and the removal of damaged RBC following transfusion, as supplementation with 5-HT improves RBC post-transfusion survival in a mouse model of blood banking.

## Introduction

Serotonin (5-hydroxytryptamine or 5-HT) is a monoamine originally purified from blood as a vasoactive agent and known for its role in the coagulation process. Since the identification of 5-HT, its role as a neurotransmitter in the central nervous system has attracted considerable attention; yet, increasing evidence substantiates the role of 5-HT in the regulation of important nonneuronal functions. Several receptors, encoded by at least 15 different genes, which are divided into seven subfamilies (5-HT_1–7_), are activated by 5-HT and are responsible for its effects [1]. Availability of 5-HT depends on the expression of the enzyme tryptophan hydroxylase (TPH), which catalyzes the first and rate-limiting step in its biosynthesis [2]. TPH is found only in 5-HT-producing cells, and exists in two forms, one of which is found predominantly in the central nervous system (TPH2) while the other (TPH1) is in peripheral tissues [3], [4]. We and others previously showed that targeted disruption in mice of the *TPH1* gene results in very low levels of circulating 5-HT, but normal levels of 5-HT in the brain [3], [4]. In depth analysis of the TPH1 knockout mouse revealed that they develop a phenotype of macrocytic anemia resulting from both an ineffective erythropoiesis in the bone marrow and a reduced half-life of their circulating red blood cells (RBC) [5].

In humans, the standard maximum duration of routine RBC storage as approved by the US and European legislation is of 42 days. RBC storage solutions are minimal in their composition, and were mostly designed to maintain RBC in an adequate metabolic state. Indeed, it has long been known that aging of RBC in storage solutions is associated with an increase of potassium, lactate, free hemoglobin levels, and a decrease of glucose, 2,3-disphosphoglycerate (2,3-DPG) and ATP levels [6]. These solutions, however, do not completely protect the banked RBC as they undergo significant morphological and molecular changes during storage. These changes, collectively termed “RBC storage lesions”, can be catalyzed by oxidation, and include a gradual degradation of membrane proteins such as Band-3 [7]–[12]. Accelerated senescence due to oxidation is not limited to storage conditions as it may also be at play during the normal aging process *in vivo*. Hence, compromised protection from oxidative damage or accumulation of intracellular reactive oxygen species (ROS) results in a shortened lifespan of RBC and may lead to anemia [13]–[17]. Consistent with this, RBC contain a strong arsenal of antioxidant enzymes that protect the cells against ROS, including catalase, superoxide dismutase and glutathione peroxidase [18], and a number of circulating antioxidant small molecules such as vitamin E, β-carotene and glutathione, which are present in plasma and may contribute to the antioxidant defense of the organism [19], [20].

In this report, knowing that 5-HT is a physiological molecule present in circulation, we show that 5-HT acts as an antioxidant and is critical for RBC survival in mouse. In addition, we provide evidence for a link between oxidation and the removal of damaged RBC following transfusion as supplementation of RBC storage solution with 5-HT improves post-transfusion RBC survival in a mouse model of blood banking.

## Methods

### Mouse transfer experiments

Targeted mutagenesis of the *TPH1* gene was previously described [3]. Animals on a C57BL/6J background and 6–8 weeks of age were used. Donor blood was biotinylated by intravenous injection of 50 mg/kg of (+)-biotin-N-hydroxysuccinimide ester (Sigma), and collected with EDTA-coated pipettes. Biotinylated RBC were purified using Histopaque 1083 (Sigma) and transfused into WT or TPH1^−/−^ recipient mice on day 0. Percentage of biotinylated cells in blood of recipient mice was determined by flow cytometry (FACS).

### Mouse *in vitro* experiments

Blood was obtained by orbital enucleation and collected in an EDTA tube. RBC were purified using Histopaque 1083. The hematocrit of each sample was adjusted to 5% by adding RPMI supplemented with 20 mM hepes (final osmolality of 315mOsm/Kg), using an electronic hematology particle counter (Melet Schloesing Laboratories), and stored in sealed 500 µl tubes at 4°C until analysis. Hemolysis was evaluated using Drabkin’s reagent, and phosphatidylserine exposure was measured by FACS using APC-annexin V. Tryptophan, 5-hydroxytryptophan, 5-HT, melatonin, trolox and BIMU-8 were from Sigma. The 5-HT receptor agonists, 8-OH DPAT, GR 46611, PNU 22394, WAY 208466 and LP-12 were from Tocris Bioscience. Binding experiments were performed as previously described [21].

### Total antioxidant capacity of mouse plasma

Mouse blood was obtained by cardiac puncture in a heparinized tube and centrifuged for 5 min at 500 g. Plasmas were submitted to the “total antioxidant status” test following the manufacturer’s instructions (Randox Laboratories).

### Mouse model of blood banking and transfusion

Blood was collected in a CPDA-1 solution (14%) and leukoreduced using a neonatal high-efficiency leukoreduction filter for RBC (Pall). Blood was transferred into sealed 500 µl tubes, supplemented with 0, 10, 30 or 100 µM of 5-HT and stored at 4°C. At regular intervals, stored RBC were stained with carboxyfluorescein diacetate succinimidyl ester (CFDA-SE), washed twice with PBS and transfused into recipient mice. Recipient mice were bled 5 min (max) and 24h (residual) post-transfusion, and the percentage of donor CFDA-SE^+^ RBC was measured by FACS. The 24h post-transfusion survival was determined by the ratio of the percentage of CFDA-SE^+^ RBC at 24h divided by the percentage of CFDA-SE^+^ RBC at 5 minutes.

### Ethics Statement

All animal experiments were conducted in accordance with the French regulations and the European Union guidelines for the care and use of animals. Protocols were approved by the Direction de Services Vétérinaires, the competent French veterinarian agency under agreement B75-15-15.

## Results

**Figure 1:**
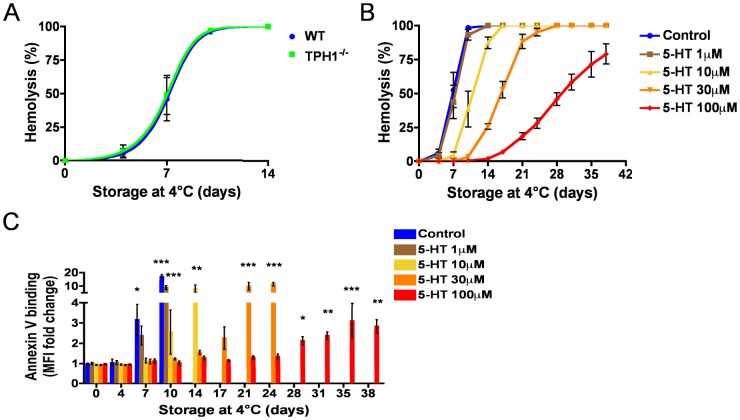
Serotonin (5-HT) protects mouse RBC from senescence *in vitro*. Mouse RBC from WT or TPH1^−/−^ mice were incubated at 4°C and their survival assessed by measuring hemolysis (A). Alternatively, mouse RBC from WT mice were incubated with increasing doses of 5-HT and their survival (B) and annexin V binding capacity (C) were evaluated. Data in each panel are presented as mean ± SEM of at least 4 independent experiments. In (C), *P<0.05; **P<0.01; ***P<0.001 when compared to value at day 0 in each group by one-way ANOVA and Dunnet’s post-hoc test.

**Figure 2:**
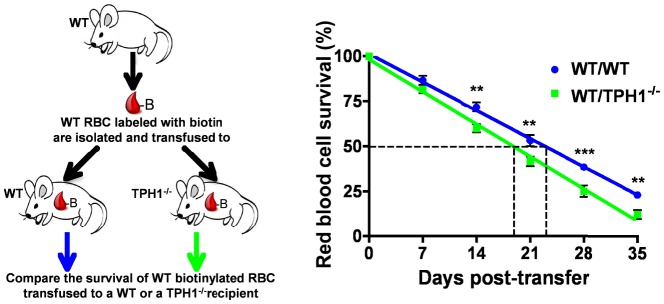
Serotonin (5-HT) protects mouse RBC from senescence *in vivo*. Schematic diagram (left) of the transfer experiments conducted to evaluate the *in vivo* survival of WT RBC (right) in either a normal (WT, n = 12) or a 5-HT-depleted environment (TPH1^−/−^, n = 11). **P<0.01; ***P<0.001 by two-way ANOVA and Bonferroni’s post-hoc test.

Previous *in vivo* data demonstrated that RBC from 5-HT-deficient mice were more sensitive to macrophage phagocytosis and had a shortened *in vivo* half-life [5]. To establish whether the observed reduced half-life in 5-HT-deficient mice (TPH1^−/−^) is an intrinsic characteristic, we compared *in vitro* survival of RBC isolated from TPH1^−/−^ as compared to wild-type (WT) mice. RBC were incubated at 4°C and their survival was assessed during a 14 day-period by measuring hemolysis. As shown in Figure 1A, the RBC survival curve was comparable for both genotypes with a half-life of 7.2 days. We thus hypothesized that 5-HT exerts an extrinsic protective effect on RBC and to demonstrate the effect, we first used an *in vitro* system in which WT mouse RBC were incubated in a medium supplemented with increasing doses of 5-HT. A striking dose-dependent protective effect of 5-HT on mouse RBC was observed (Figure 1B) as 5-HT supplementation extended the half-life of RBC from 10.7 days (10 µM) to 16.4 days (30 µM), and 29.1 days at the highest dose used (100 µM). Next, we assessed the appearance of phosphatidylserine (PS) at the surface of RBC, as measured by annexin V binding to non-hemolysed RBC of normal forward-scatter and side-scatter characteristics (Figure 1C). Figure 1C illustrates that increasing doses of 5-HT delayed the appearance of PS at the surface of RBC, demonstrating that 5-HT supplementation prevented the appearance of this senescence marker. In light of these *in vitro* data, we hypothesized that the decreased *in vivo* half-life of RBC from 5-HT-deficient mice was due to a suboptimal environment resulting from the lack of 5-HT. We thus performed *in vivo* experiments in which RBC were transferred in an environment with very low levels of 5-HT (Figure 2 left panel). More precisely, WT mouse RBC were isolated, labeled with biotin and their survival rate were compared following transfusion either to a WT or a 5-HT-deficient recipient. Figure 2 (right panel) indicates that WT RBC circulating in a 5-HT-deficient environment *in vivo* have a diminished half-life when compared to RBC transferred to a WT recipient with normal physiological 5-HT level (18.8 days *vs* 23.0 days). This *in vivo* result is in agreement with our *in vitro* data, and demonstrates that 5-HT protects RBC through an extrinsic effect.

**Figure 3:**
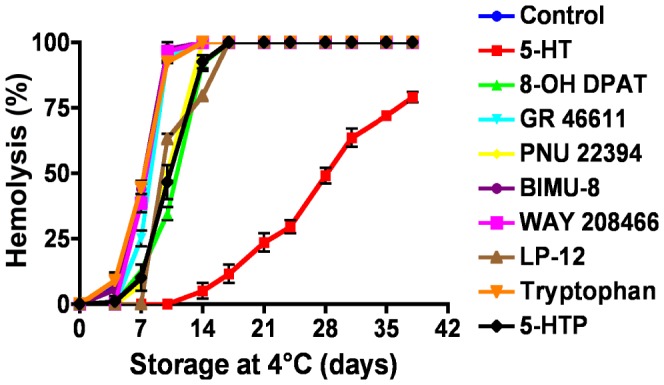
Serotonin (5-HT) precursors and agonists have no protective effect on mouse RBC *in vitro*. Mouse RBC from WT mice were incubated at 4°C and their survival assessed by measuring hemolysis. The 5-HT precursors tryptophan, 5-hydroxytryptophan (5-HTP) or agonists for the 5-HT1A (8-OH DPAT), 5-HT1B/1D (GR 46611), 5-HT2 (PNU 22394), 5-HT4 (BIMU-8), 5-HT6 (WAY 208466) or the 5-HT7 receptors (LP-12) could not mimic the protective effect of 5-HT. All molecules were tested at a concentration of 100 µM. Data are presented as mean ± SEM of 3 independent experiments.

Classically, 5-HT exerts its effect by binding to specific membrane receptors and to determine whether the 5-HT protective effect on RBC is receptor-mediated, we conducted a thorough analysis of the 5-HT receptor repertoire expressed by mouse RBC using radioactive binding assays. To insure the purity of the studied material, RBC were sorted electronically, and Ter119^+^/CD41^−^ cells were selected to specifically eliminate platelets contaminants, known to express some 5-HT receptor subtypes (5-HT_2A_ [22] and 5-HT_1_-like [23]). None of the molecules tested could bind RBC membranes, showing that mouse RBC do not express 5-HT receptors (Table 1). Moreover, *in vitro* incubation of mouse RBC in the presence of different 5-HT receptor agonists and 5-HT precursors did not result in a protective effect (Figure 3). These data indicate that the protective effect is not mediated through activation of the classical serotonergic pharmacological pathway.

**Table 1:** Mouse red blood cells do not express 5-HT receptors.

Radioactive ligand used	Receptor type	Ligand binding to red blood cells (fmoles/mg protein)
^3^H-8OHDPAT	5-HT_1A_	< 10
^125^I-GTI	5-HT_1B_, 5-HT_1D_	< 0.5
^125^I-LSD	5-HT_1F_, 5-HT_5_, 5-HT_6_	< 0.5
^125^I-DOI	5-HT_2A_, 5-HT2_B_, 5-HT_2C_	< 0.5
^3^H-ramosetron	5-HT_3_	< 10
^125^I-SB207710	5-HT_4_	< 0.5
^3^H-SB269970	5-HT_7_	< 10

^+^CD41^−^ cells isolated from mouse blood. Each binding experiment was conducted on 4 separate samples. Binding was performed on membranes of sorted Ter119

**Figure 4:**
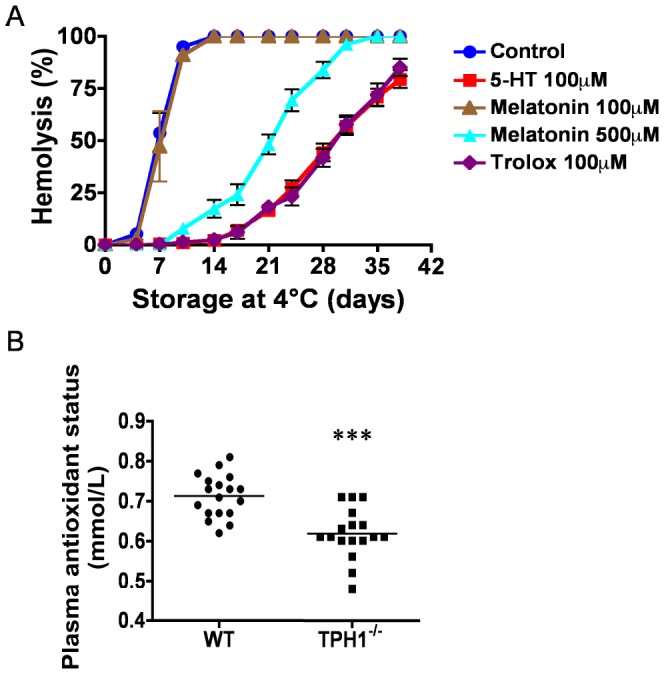
Serotonin (5-HT) contributes to the antioxidant potential of plasma and its protective effect on RBC can be replicated by antioxidants. Mouse RBC from WT mice were incubated at 4°C with the antioxidants trolox or melatonin and their survival assessed by measuring hemolysis (A). In (B), total antioxidant test was conducted on either normal (WT, n = 18) or 5-HT-deficient plasma (TPH1^−/−^, n = 17) to measure the plasma antioxidant capacity. Data are presented as mean ± SEM. ***P<0.001, by student t test.

An alternative mechanism for the 5-HT protective action might be through its antioxidant properties. Indeed, 5-HT has been suggested to be a potent antioxidant in different *in vitro* tests [24]. To test this possibility, we supplemented mouse RBC incubated *in vitro* with trolox, a vitamin E analog and well-known antioxidant, or with melatonin, a monoamine derived from 5-HT with recognized antioxidant properties. Our results show that trolox was as effective as 5-HT to prevent hemolysis (Figure 4A). Addition of melatonin also delayed hemolysis, although a high dose was necessary to observe any protective effect. These data confirm that oxidative damage is an important cause of hemolysis in our model. To further attest that circulating 5-HT acts as an antioxidant *in vivo*, we compared the total antioxidant capacity of WT *vs* TPH1^−/−^ plasma, the latter containing very low levels of 5-HT. As expected, the antioxidant capacity of TPH1^−/−^ plasma was significantly reduced (p<0.0001; Figure 4B). Altogether, the data support the view that 5-HT contributes significantly to the antioxidant capacity of mouse plasma and protects RBC from oxidant-induced senescence both *in vitro* and *in vivo*.

**Figure 5:**
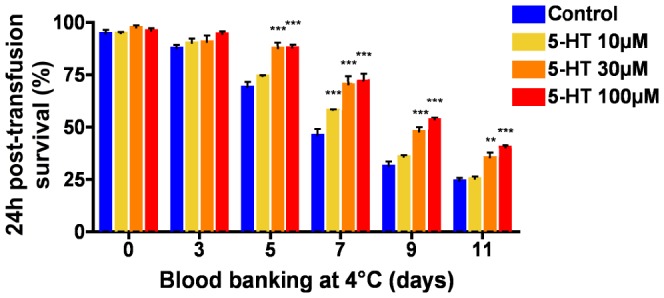
Serotonin (5-HT) supplementation improves 24h post-transfusion survival of RBC in a mouse model of blood banking. Donor blood was collected in CPDA-1, leukoreduced and stored in sealed tubes in CPDA-1 supplemented or not with increasing doses of 5-HT. After storage, RBC were stained with CFDA-SE, transfused to recipient mice and their 24h post-transfusion survival was assessed by flow cytometry. Data are presented as mean ± SEM (n = 4–7). **P<0.01, ***P<0.001 by two-way ANOVA and Bonferroni’s post-hoc test.

Oxidation has been identified as a major cause of the hypothermic storage lesions during blood banking [7]–[9], [11]. The likelihood that 5-HT exerts the same protective effect on human RBC in conventional blood bank storage conditions would be of major significance, both for clinical applications in transfusion medicine and for the elaboration of new storage solutions. When blood is donated, it is stored according to defined regulations. One important criterion used by the Regulatory Agencies before approval of a RBC storage system, is a measure of post-transfusion survival that is a measure of *in vivo* viability, which reflects the quality of stored RBC [25]. Recently, inventive efforts to study the post-transfusion survival of RBC in model animal systems have revealed important information related to the storage lesion and transfusion-related reactions [26]–[28]. Using such an established mouse model of blood banking [27], [28] we evaluated the impact of 5-HT supplementation on the 24h post-transfusion survival of stored RBC. In order to closely mimic the aging process undergone by human RBC during hypothermic storage, mouse RBC were collected in the conventional human storage solution CPDA-1, stored at 4°C after removal of the leukocytes by filtration, stained with CFDA-SE and transfused to a recipient to follow their post-transfusion survival. Results shown in Figure 5 are presented as the percentage of transfused RBC still in circulation 24h after transfusion, following storage at 4°C. Supplementation of CPDA-1 with 5-HT led to a dose-dependent increase in the 24h post-transfusion survival of mouse RBC. Following 7 days of storage, the 24h post-transfusion survival was increased by 26% when stored in the presence of 10 µM 5-HT and by more than 50% with 30 µM and 100 µM 5-HT. These data further show the protective effect of 5-HT and support the view that 5-HT extends the viability of stored RBC by preventing oxidation-related storage lesions.

## Discussion

The mouse data presented here reveal that 5-HT acts as a circulating antioxidant to protect RBC from senescence. This protection is effective *in vitro* and *in vivo* during aging of RBC as addition of 5-HT to stored RBC extends their shelf life, while reduction of 5-HT circulating levels decreases both the antioxidant capacity of plasma and the RBC lifespan. In addition, using a mouse model of blood banking, we show that 5-HT supplementation to stored RBC improves their post-transfusion survival.

The reduced half-life of circulating RBC previously observed in TPH1^−/−^ mice [5] is not due to an intrinsic defect of RBC, but to the reduced level of circulating 5-HT. Indeed, even though TPH1^−/−^ RBC expose more PS and are more sensitive to macrophage phagocytosis [5], they have a hemolysis curve comparable to the one of WT RBC when isolated *in vitro*. More importantly, the transfer of WT RBC to a TPH1^−/−^ recipient reduces their *in vivo* half-life, showing that circulation in a 5-HT-deficient environment reduces their lifespan. Also, 5-HT addition to a RBC storage medium *in vitro* delays hemolysis and the appearance of the senescence marker, PS. Even tough the concentration of 5-HT needed to significantly extend RBC half-life *in vitro* (10 µM) is close to the detected 5-HT level of 6.4 µM in whole blood of WT mice (0.3 µM in TPH1 KO) [29], it is superior by one log to its detected level of 15.3 nM in platelet poor plasma (6nM in TPH1 KO). 5-HT of the platelet poor plasma has always been considered the “biologically active” compartment since most of blood 5-HT is stored in dense granules of the platelet compartment. At this moment, we cannot exclude that part of the reduced survival of RBC in the 5-HT-deficient environment could be due to a macrophage phenotype of the TPH1 KO mouse. Along that line, it was recently shown that 5-HT could modulate macrophage polarization *in vitro* [30]. Further experiments would be needed to elucidate this possibility but, nevertheless, the decreased RBC half-life and decreased circulating 5-HT level *in vivo* is in agreement with our *in vitro* results showing a protective role of 5-HT on mouse RBC.

The protective effect played by 5-HT is not mediated through activation of a classical pharmacological pathway as no 5-HT receptors were detected on isolated RBC and no 5-HT agonists could mimic its effect. The protective effect of 5-HT could, however, be replicated by trolox, a vitamin E analog, suggesting that the antioxidant capacity of 5-HT is involved. In fact, 5-HT was previously suggested to prevent lipid peroxidation of platelet membranes *in vitro* [31]. Similarly, the hydroxyl group of trolox or vitamin E acts as a potent peroxyl radical scavenger to prevent the propagation of free radicals in membranes and plasma lipoproteins [32], [33].

Numerous natural substances are recognized for their efficiency as oxidant scavengers in fruits and vegetables. These natural compounds usually have a chemical structure encompassing reductive properties: thiol (glutathione) or phenol functions (vitamin E, resveratrol), flavonoids (quercetin), but also heterocyclic structures bearing the indole nucleus such as tryptophan derivatives [34]. In this capacity, 5-HT is synthesized in rice leaves upon senescence, which is delayed through the antioxidant activity of 5-HT [35]. Indeed, comparison of transgenic rice plants producing either high or low levels of 5-HT showed a phenotype of delayed senescence in the 5-HT-rich plant but accelerated senescence in the 5-HT-poor variety [35]. However, such an antioxidant role for 5-HT has never been described in the animal kingdom as most of the research toward indole-based molecule has been oriented toward the study of melatonin [36]. Our demonstration that the total antioxidant capacity of TPH1^−/−^ plasma was reduced when compared to WT shows that 5-HT significantly contributes to the antioxidant potential of plasma. Oxidative stress is an important factor contributing to reduction of the RBC lifespan, and presence of intracellular and extracellular antioxidants is necessary to prevent oxidative damage. For example, deletion of superoxide dismutase 2 or inactivation of antioxidant enzymes containing a peroxidatic cysteine (peroxiredoxin I and II) causes hemolytic anemia [14], [17], [37]. Likewise, the half-life of circulating RBC is reduced in the Foxo3 knockout mice, due to a diminished expression of ROS scavenging molecules [16]. More importantly, clinical deficiency of vitamin E in humans can cause varying degrees of hemolysis. For instance, pediatric patients with cystic fibrosis and vitamin E deficiency develop hemolytic anemia [19], [20].

On the basis of the data presented, there may be post-transfusion RBC survival advantages in humans when supplementing RBC concentrates with an antioxidant such as 5-HT. This is supported by the fact that 5-HT supplementation improves post-transfusion survival in our mouse model of blood banking. In this established model [27], [28], mouse RBC are directly collected in the conventional human storage medium CPDA-1 and leukoreduced using conventional filters, allowing for storage conditions similar to those used during conventional blood banking. This result is crucial since it confirms that 5-HT is not only protecting RBC from hemolysis *in vitro*, but that it allows survival in the more challenging environment of the *in vivo* circulation. The measure of the post-transfusion survival is thus an assessment of all storage lesions related to the RBC rheology that occur during hypothermic storage. Also, an important requirement for the approval of new storage solutions is based on an *in vivo* survival rate of the transfused cells of more than 75%, at 24 hours after transfusion, at the end of the RBC storage shelf life [38], [39].

The oxidative injury occurring during storage is thought to be responsible for the progressive decline in deformability of the stored RBC and gradual degradation or aggregation of membrane proteins such as Band-3 [7]–[10], [40], [41]. Both these modifications can lead to the removal of the transfused cells shortly after transfusion, thus reducing the efficiency of the process and putting the patient at risk. Indeed, there is an ongoing debate in the transfusion community as the results of some clinical studies confirm the link between RBC storage lesions (transfusion of old RBC) and the negative clinical outcome of transfused patients [42]–[47]. Thus, an approach to counteract the oxidative stress would improve the quality of stored RBC, extend storage time, and potentially reduce transfusion-related adverse reactions. Along this line, Yoshida's group was able to diminish storage lesions by eliminating oxygen from the storage bag [48], [49]. However, it was recently shown that anaerobic storage could impair the RBC metabolic capacity to cope with oxidative stress [50]. Hence, supplementation with an antioxidant (such as 5-HT) would be an attractive strategy to slow the oxidative-accelerated aging process observed during hypothermic storage. Still, care should be taken in the choice of the supplemented antioxidant to avoid any side effect associated with its transfusion to a patient. Taking in consideration the increasing number of prescribed pro-serotonergic agents, transfusion of 5-HT may trigger a serotonin syndrome [51] and, as such, identification of an antioxidant with pre-transfusion protective properties and without post-transfusion risks should be the goal of future research. Knowing the advantages and limitations of the mouse model [52], the beneficial effects of antioxidant supplementation to storage solutions on post-transfusion RBC survival demonstrated in mouse will need to be replicated in humans.
